# 

**Published:** 1898-09

**Authors:** 


					﻿Vol. XVIII.
SEPTEMBER, 1898.
NO. 9?
THE
HOMEOPATHIC PHY^ICIAK
A Monthly Journal of Homoeopathic Materia Medica
and Clinical Medicine.
“ He is a freeman whom truth makes free, and all are slaves beside.”
“Seek the Truth: come whence it may, cost what it will."
EDITED AND PUBLISHED BY
WALTER ML. JAMES, ML. D.
PHILADELPHIA :
Ko. 1231 LOCUST STZREET.
Yearly Subtcripiicn, $2.50, invariably tn advance. Single nambertj 95 etc.
Entered at the Philadelphia Poat-Offiee aa Beoond*01aaa Mail Matter.
				

